# Supplementary material for: Challenges with pediatric antiretroviral therapy administration: Qualitative perspectives from caregivers and HIV providers in Kenya
Source: PLoS One. 2024 Jan 9;19(1):e0296713. doi: 10.1371/journal.pone.0296713 (PMC10775971; doi:10.1371/journal.pone.0296713)
Supplement: Attachment — Submitted filename: Response to review comments_3.docx [file pone.0296713.s002.docx]

Reviewer #2: ABSTRACT
1. In your abstract, as you report findings, highlight what was reported by both groups (caregivers, healthcare providers) and then what was unique to each group. This is still pending.

We have specified which results came from which participant group.

2. Consider having a conclusion, not a discussion section in the abstract.

We have changed discussion to “conclusion” in the abstract.

METHODS
3. Your study used other languages besides English. Add information on informed consent, translation and associated consenting documents. This is still pending.

We have specified that: “Participants provided written informed consent in compliance with IRB approvals at the University of Kansas Medical Center (STUDY00147024) and University of Nairobi (P457/06/2021). Caregiver informed consent documents and FGD guides were available in English, Luo, and Swahili and all documents and translations were approved by the IRB at the University of Nairobi. Provider informed consent documents and interview guides were available in English only, as all healthcare providers were fluent in English. During the informed consent process, the interviewer explained the purpose of the study, risks, and benefits of the study, and emphasized that study participant was optional. Immediately after informed consent was provided but prior to the focus groups or KIIs, surveys with each participant were conducted to capture socio-demographics characteristics of providers and caregivers. Informed consent interviews and surveys occurred in a private area of the hospital and were conducted by a research assistant in English, Swahili, or Luo, per the participant’s preference. “ We have added a line that specifies caregiver documents were available in 3 languages and that all documents and translations were approve by the IRB at a local institution.

4. It is still not clear which findings come from which of the 9 FGDs and/or which of the KIIs. I suggest you get 3 index refereed articles to guide you in your data analyses and reporting. You need to:
*Run analysis for the 9 FGDs
*Run analysis for each of the 30 KIIs
*Run a summary that shows your findings by each of the 3 sites
*It would also help to run a summary that shows codes/sub codes by caregiver characteristics
Then do a comparative analysis. I find it easier to use a visual- a summary matrix during my data management and analysis. For such a study, it would have 39 columns to capture all the codes/sub-codes from all the data collection sessions. It makes comparison easier.

The objective of this study was to assess general challenges to pediatric ART in Kenya to inform development of a product that could overcome these challenges. While we included multiple caregiver types to represent the range of caregivers who provide care to children living with HIV, we did not intend to analyze differences between caregiver types. That said, there was great consensus across the concerns raised. We feel that such a quantitative approach to this qualitative study is unnecessary given the consensus.

5. Edit grammar in this section.

We have edited the grammar to standard English.

RESULTS
6. Ensure all content provided in Table 1 is reported in narrative format. This is still pending. This guideline applies where all tables are concerned.

The paragraph proceeding the tables summarizes key items in the table without being redundant of the information in the table. We feel that adding more descriptive text would not add meaningful content to the manuscript and would be duplicative of the information provided in the table.

7. You continue to provide sections of transcripts as data findings. This is still pending.

Per the COREQ guidelines, which we have attached as a supplementary material and complies with journal requirements, item 29 requires participant quotations to highlight themes and findings. We have opted to retain these quotations as required by journal guidelines and as the accepted practice in qualitative data presentation.

8. Do findings differ by sites? FGDs? KIIs? Demographics - especially care giver characteristics?

Per response above: The objective of this study was to assess general challenges to pediatric ART in Kenya to inform development of a product that could overcome these challenges. While we included multiple caregiver types to represent the range of caregivers who provide care to children living with HIV, we did not intend to analyze differences between caregiver types. That said, there was great consensus across the concerns raised. We feel that such a quantitative approach to this qualitative study is unnecessary given the consensus.

DISCUSSION
9. This section has improved. Thank you
Once you revise the results section, refine the discussion and conclusion sections.

We have opted not to revise the results section, as discussed above.

CONCLUSION
10. Add a conclusion in this manuscript.

We have added a conclusion to this manuscript.
